# Microwave Antenna Sensing for Glucose Monitoring in a Vein Model Mimicking Human Physiology

**DOI:** 10.3390/bios15050282

**Published:** 2025-04-30

**Authors:** Youness Zaarour, Fatimazahrae El Arroud, Tomas Fernandez, Juan Luis Cano, Rafiq El Alami, Otman El Mrabet, Abdelouheb Benani, Abdessamad Faik, Hafid Griguer

**Affiliations:** 1 Microwave Energy Sensing (MES), DICE—Digital Innovation Center of Excellence, University of Mohammed VI Polytechnic, Benguerir 43152, Morocco; fatima.arroud@um6p.ma (F.E.A.); rafiq.elalami@um6p.ma (R.E.A.); hafid.griguer@um6p.ma (H.G.); 2 Departamento de Ingeniería de Comunicaciones, Universidad de Cantabria, 39005 Santander, Spain; tomas.fernandez@unican.es (T.F.); juanluis.cano@unican.es (J.L.C.); 3 Intelligent System Design Laboratory (ISD), Faculty of Science, Abdelmalek Essaadi University, Tetuan 93000, Morocco; oelmrabet@uae.ac.ma; 4 Oncovirology Laboratory, Institut Pasteur du Maroc, 1, Place Louis Pasteur, Casablanca 20360, Morocco; abdelouaheb.benani@pasteur.ma; 5 Laboratory for Inorganic Materiels for Sustainable Energy Technologies (LIMSET), University of Mohammed VI Polytechnic, Benguerir 43152, Morocco; abdessamad.faik@um6p.ma

**Keywords:** tissue-mimicking phantom, non-invasive glucose monitoring, veins simulation, electromagnetic sensing, miniature patch antenna

## Abstract

Non-invasive glucose monitoring has become a critical area of research for diabetes management, offering a less intrusive and more patient-friendly alternative to traditional methods such as finger-prick tests. This study presents a novel approach using a semi-solid tissue-mimicking phantom designed to replicate the dielectric properties of human skin and blood vessels. The phantom was simplified to focus solely on the skin layer, with embedded channels representing veins to achieve realistic glucose monitoring conditions. These channels were filled with D-(+)-Glucose solutions at varying concentrations (60 mg/dL to 200 mg/dL) to simulate physiological changes in blood glucose levels. A miniature patch antenna optimized to operate at 14 GHz with a penetration depth of approximately 1.5 mm was designed and fabricated. The antenna was tested in direct contact with the skin phantom, allowing for precise measurements of the changes in glucose concentration without interference from deeper tissue layers. Simulations and experiments demonstrated the antenna’s sensitivity to variations in glucose concentration, as evidenced by measurable shifts in the dielectric properties of the phantom. Importantly, the system enabled stationary measurements by injecting glucose solutions into the same blood vessels, eliminating the need to reposition the sensor while ensuring reliable and repeatable results. This work highlights the importance of shallow penetration depth in targeting close vessels for noninvasive glucose monitoring, and emphasizes the potential of microwave-based sensing systems as a practical solution for continuous glucose management.

## 1. Introduction

Diabetes is a chronic metabolic disorder that affects over 537 million adults worldwide, with the number projected to rise to 783 million by 2045. It remains a leading cause of morbidity and mortality, contributing to an estimated 1.5 million deaths annually, either directly or through associated complications such as cardiovascular disease and kidney failure. Effective management of diabetes hinges on regular monitoring of blood glucose levels to prevent long-term complications and improve patient outcomes [1,2,3,4].

Despite its importance, blood glucose monitoring is predominantly performed using invasive methods such as finger-prick tests with blood glucose meters (BGMs). While these methods are accurate and widely available, they are also painful, inconvenient, and costly, often leading to poor adherence among patients [5,6]. For instance, frequent testing requires the continuous purchase of test strips, which can cost patients hundreds of dollars annually. This financial burden is compounded by the discomfort associated with pricking the skin multiple times a day [7]. Advancements in continuous glucose monitoring (CGM) technologies have introduced alternatives that reduce the invasiveness of traditional methods. However, as of 2024, only 6.3% of individuals with diabetes globally utilize CGM devices, primarily due to their high cost and occasional accuracy issues. Consequently, approximately 93.7% of people with diabetes still rely on traditional BGMs for monitoring their glucose levels [8,9,10]. These statistics underscore the urgent need for innovative, noninvasive, and cost-effective solutions for glucose monitoring [11]. The prevalence of traditional BGM usage underscores the necessity for developing noninvasive glucose monitoring technologies that can offer a less intrusive and more comfortable alternative for patients [12]. Microwave-based sensing technologies offer an exciting, noninvasive, and affordable way to monitor glucose levels in real time [13,14]. These technologies work by analyzing how electromagnetic waves interact with the body’s tissues in different locations, which change depending on glucose levels [15]. This interaction is often characterized using S-parameters, or scattering parameters, which describe how signals are transmitted ($S_{21}$) or reflected ($S_{11}$) in a system. Some approaches focus on the $S_{21}$ response to measure how signals pass through tissues [16,17], while others examine $S_{11}$ to analyze reflected signals [18,19,20]. Each method offers distinct advantages depending on how the sensors are set up, giving researchers flexibility in designing systems tailored for specific applications. While promising, testing these technologies on humans during development poses challenges. Studies may lack prior knowledge on the dielectric properties of tissue or blood, leading to inconsistent results [21]. Experiments can be costly, impractical, and complicated by the body’s diverse and variable tissue properties. To overcome these challenges, researchers have developed tissue-mimicking phantoms that replicate the dielectric properties of tissues such as skin, fat, blood, and muscle. These phantoms enable controlled testing and optimization of microwave sensors by simulating glucose-induced dielectric variations in the body [22,23,24,25,26].

However, many studies rely on simplified models of human tissues, often representing them as stacked homogeneous layers. While these models are helpful for initial investigations, they fall short of capturing the true complexity of human anatomy. For instance, ref. [18] used PDMS (polydimethylsiloxane)-based microfluidic vascular phantoms designed from angiograms of hand arteries to test glucose sensors. Although this approach demonstrates sensor responsiveness to glucose variations, PDMS does not replicate the unique structural and dielectric properties of human skin, which could affect the accuracy of the results. Moreover, in [24] the researchers took a different approach by mimicking skin, fat, muscle, and blood to replicate the dielectric properties of real tissues. However, in order to simulate glucose concentration changes within the blood layer, they replaced the blood layer for each glucose level. While this method resulted in low impedance error, it may still introduce inaccuracies in final glucose concentration measurements due to the disruption of a stable and continuous testing environment. Finally, there is a lack of detailed methodologies in the literature for mimicking realistic blood glucose level (BGL) variations in phantoms. Developing phantoms that are stable, accurate, and capable of representing realistic vein structures is essential to ensure reliable evaluations of sensor performance and advance these technologies toward clinical viability.

To the best of our knowledge, this study is one of the first to extensively investigate the use of radio frequency (RF) techniques for replicating and monitoring changes in blood glucose levels (BGL) within veins under controlled conditions that simulate the properties of human skin. This work incorporates empty channels within a skin phantom designed by using embedded tubes to replicate veins. These channels are modeled on angiogram images of the upper hand, which specifically highlight the brachial veins. This approach ensures that the phantom closely resembles the anatomical structure of human veins. For this proof-of-concept, we chose to replicate only the skin in order to establish a foundational framework for future studies. In addition, the complexity of the veins is represented by two parallel empty cylinders, simulating simplified vein structures for initial testing. These channels are then filled with glucose solutions of varying concentrations, enabling dynamic glucose variations to be studied in a continuous monitoring context. Building on phantoms with dielectric properties similar to human tissues, this approach provides a more realistic representation of physiological conditions that can enhance experimental setups and advance RF-based glucose monitoring.

A miniature patch antenna optimized to operate at 14 GHz and achieve a penetration depth of approximately 2 mm was carefully designed and fabricated to ensure reliable performance when in direct contact with a layered skin tissue phantom. To validate its functionality, we conducted both simulations and experiments involving the injection of glucose solutions with varying concentrations into the phantom. Glucose solutions prepared using D-(+)-Glucose at concentrations ranging from 60 mg/dL to 200 mg/dL were injected into the same layer using a syringe. To maintain consistency, the system remained stationary throughout and the phantom layer was thoroughly cleaned between measurements in order to prevent cross-contamination. Section 2 details the fabrication of tissue mimicking phantoms, including preparation recipes and dielectric property measurements, as well as the simulation of a miniature patch antenna. Section 3 presents the experimental results and analysis, highlighting the antenna’s performance and sensitivity to varying glucose concentrations. Finally, Section 4 provides the conclusions.

## 2. Materials and Methods

**Figure 1:**
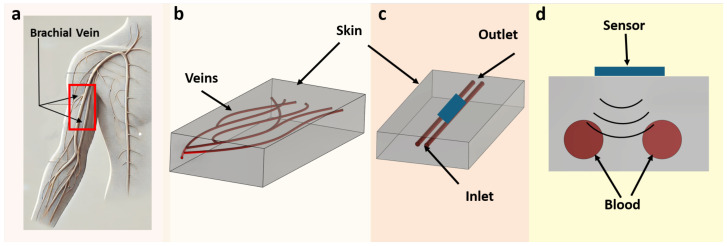
Schematic representation of vein-embedded skin phantom for RF-based glucose monitoring: (**a**) angiogram image of the upper hand; (**b**) design of 3D visualization of complex vein structures embedded within a skin phantom; (**c**) simplified representation of blood, illustrated with two straight veins; (**d**) cross-sectional view of blood veins and sensor interaction.

This section outlines the design and methodology developed to evaluate the proposed microwave-based glucose monitoring system. Central to this work is the development and validation of a tissue-mimicking phantom that replicates the layer of human skin. As illustrated in Figure 1, the phantom focuses on the key skin layer, where veins are primarily located within the dermis. This study specifically focuses on the skin and veins, excluding the effects of deeper tissues such as fat and muscle. Future studies could explore layered models incorporating these additional tissue types in order to evaluate the sensor’s comprehensive performance under more anatomically accurate conditions. In addition, a miniature patch antenna was developed and tested in direct contact with the phantom in order to assess its sensitivity and performance. These methodologies were carefully crafted to ensure accurate and reproducible results while closely reflecting the complexity of the physiological conditions depicted in Figure 1.

### 2.1. Phantom Design and Composition

The literature reports a wide variety of patient-specific phantom models used in biomedical applications, typically categorized as solid, semi-solid, or liquid depending on the specific requirements of the study [27,28]. In the present work, a semi-solid phantom was developed to accurately mimic the dielectric properties of human skin. The design is based on gelatin sheets, which provide a solidified structure with mechanical stability and dielectric characteristics comparable to real skin tissue. Unlike previous studies that have incorporated multiple layers to represent skin, fat, and muscle, this work simplifies the phantom to focus exclusively on the skin layer and embedded superficial veins. This approach was chosen in order to first validate the novel integration of veins within the skin and their impact on sensor performance. By limiting the penetration depth to approximately 1.5 mm, the design ensures that the antenna interacts only with the skin and blood vessels, eliminating the influence of deeper tissue layers [29]. After conducting our analysis, we observed that the antenna’s response remains largely unaffected beyond this depth, making deeper layers less critical for this initial validation. Additionally, the phantom includes channels that simulate veins. These allow for precise control of glucose concentration variations, providing a realistic and focused platform for evaluating the system’s performance. To enhance the phantom’s physiological accuracy, future work will incorporate deeper tissue layers such as fat and muscle, enabling more comprehensive assessment of sensor behavior under realistic conditions. The tissue-mimicking phantom was designed using a combination of ingredients, each of which was carefully selected to replicate the dielectric properties of human skin. Mimicking the lipid-like behavior of skin, water increases the permittivity of the phantom, while oil reduces it. Salt plays a crucial role in controlling the conductivity, while soap acts as an emulsifier, ensuring a stable and homogeneous mixture by blending water and oil. Finally, gelatin provides the necessary solidification, ensuring that the phantom maintains its mechanical stability and structural integrity. The proportions and percentages of these ingredients as originally proposed by [24] are presented in Table 1.

### 2.2. Construction of the Phantom

**Figure 2:**
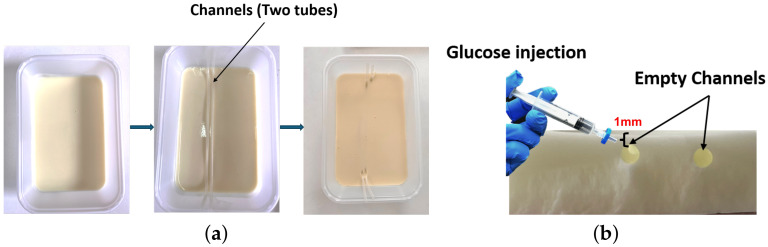
(**a**) Preparation of the skin phantom with embedded capillary-like channels and (**b**) capillary-like channels for glucose injection formed after tube removal.

As shown Figure 2, the realization of the tissue-mimicking phantom required a carefully controlled process to ensure proper mixing, solidification, and structural precision. The preparation began by dissolving gelatin sheets in heated water at 50–60 °C for 10–15 min, with continuous stirring to ensure a homogeneous solution. Oil was then gradually added to the mixture, followed by soap, which served as an emulsifier to stabilize the blend. Salt pre-dissolved in a small volume of water was then introduced to regulate conductivity.

As depicted in Figure 2a, the prepared mixture was poured into a rectangular mold to form the base of the phantom. Thin tubes with a diameter of 1.5 mm each were carefully placed within the mold to create channels that simulate blood vessels. This diameter was selected in order to closely approximate the size of superficial veins in the forearm, ensuring a realistic representation of their anatomical structure for glucose monitoring experiments. These channels were specifically designed with a 1 mm layer of gelatin above them, replicating the dermal layer of human skin and aligning with the antenna’s 1.5 mm penetration depth. The phantom was allowed to cool and partially solidify at room temperature for 1–2 h before being transferred to a refrigerator to chill for an additional 4–6 h, ensuring complete solidification and structural integrity. After the phantom was fully solidified, the tubes were gently removed, leaving well-defined empty channels, as shown in Figure 2b.

### 2.3. Preparation of Blood-Mimicking Solutions

To accurately replicate physiological glucose levels, solutions were prepared using D-Glucose dissolved in deionized water. The concentrations were 0 mg/dL (pure deionized water), 60 mg/dL, 100 mg/dL, 160 mg/dL, and 200 mg/dL, covering a range from baseline to hypoglycemic and hyperglycemic conditions. The preparation process involved precisely weighing the required amount of D-Glucose for each concentration and dissolving it in a fixed volume of deionized water. A magnetic stirrer was used to thoroughly mix the solutions, ensuring complete dissolution and homogeneity. This step was critical to maintain consistency across experiments and avoid variability in dielectric properties. Afterwards, each solution was prepared in a separate syringe to ensure precise and contamination-free measurements.

### 2.4. Antenna Design and Simulation

A miniature patch antenna was designed and optimized for operation at around 14 GHz, specifically targeting the detection of variations in glucose concentration within capillaries. A key parameter in the design is the penetration depth (δ), which ensures that the electromagnetic waves interact primarily with the dermal layer and the embedded capillary-like channels. The penetration depth is calculated using the formula

(1) $$\delta =\frac{1}{\sqrt{\pi f\mu \sigma }},$$

where *f* is the frequency of operation, $\mu =1.2566\times 10^{-6}$ H/m is the permeability of the medium, and σ=10 S/m is the conductivity of the medium, which quantifies the material’s ability to conduct electric current. Based on this equation, the theoretical penetration depth was determined to be approximately δ≈1.5 mm.

**Figure 3:**
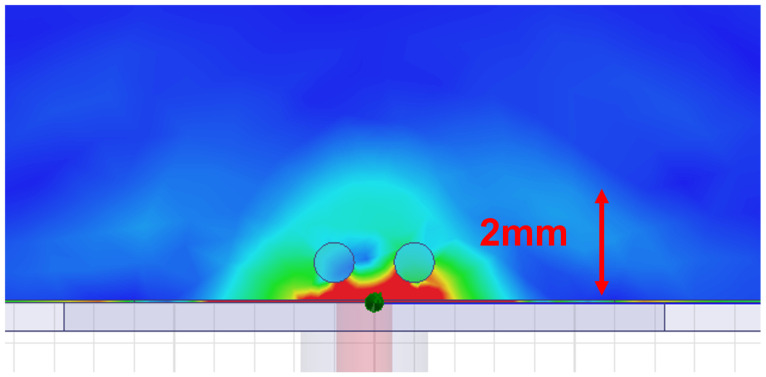
Simulated electromagnetic field distribution, showing a penetration depth of 1.5 mm into the skin phantom.

To further validate this result, a full-wave electromagnetic simulation was conducted to analyze the actual field penetration into the tissue. As shown in Figure 3, the simulated results indicate that the electromagnetic energy effectively penetrates up to 1.5 mm into the tissue, slightly exceeding the theoretically calculated depth. This slight increase can be attributed to dielectric dispersion effects and inhomogeneities in the skin phantom model, which are not fully accounted for in the simplified theoretical calculation. Despite this variation, the simulated penetration depth remains within the expected range, ensuring that the antenna’s electromagnetic fields interact efficiently with the capillary-like channels while minimizing interference from deeper tissue layers.

**Figure 4:**
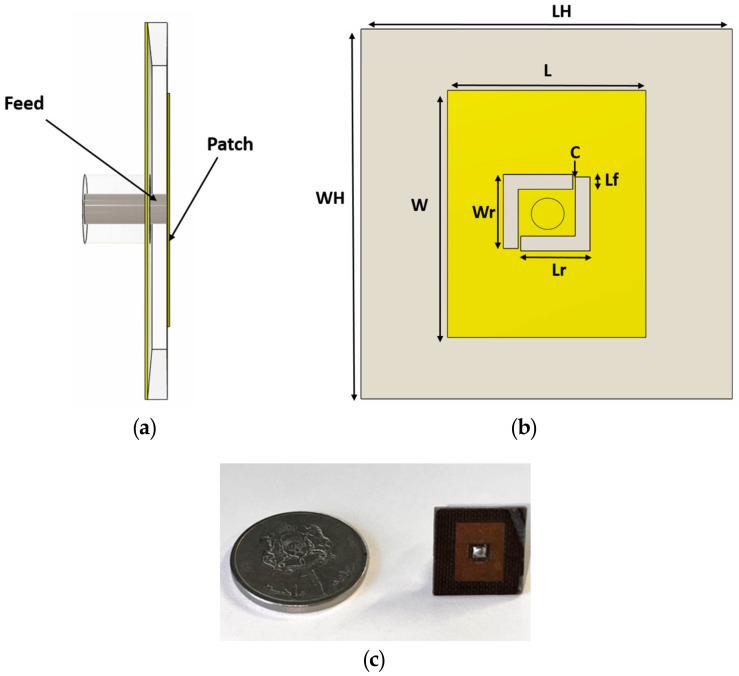
(**a**) Side view of the single-port patch antenna, (**b**) top view of the antenna geometry, and (**c**) fabricated miniature patch antenna.

The antenna was designed using a Double L resonator to achieve higher-frequency resonance around 14 GHz, enhancing its sensitivity to glucose concentration variations. Unlike a conventional rectangular patch antenna, which would face impedance mismatches when in contact with the skin phantom, the antenna structure in Figure 4 was deliberately engineered to optimize its interaction with the phantom’s electrical properties. Inspired by a U-shaped resonator, the design is divided into two smaller L-shaped resonators, allowing for greater field confinement and improved impedance matching. This configuration facilitates efficient coupling of electromagnetic energy to the superficial vein channels, ensuring that glucose-induced dielectric changes produce a significant resonance frequency shift, thereby enhancing detection accuracy. The design features a single-port configuration with the patch fed through an SMA connector, as depicted in Figure 4a. The geometry of the antenna is illustrated in Figure 4b, including critical design parameters such as the patch length (*L*), width (*W*), resonant arm dimensions ($L_{r}$, $W_{r}$), and feed gap spacing (*C*). These parameters were optimized to ensure that the antenna operates effectively at 14 GHz. This frequency was chosen due to its small penetration depth, which enables the antenna to interact specifically with the vein located approximately 3 mm beneath the surface to ensure targeted sensing. To further enhance the antenna’s performance, it was fabricated using a TACONIC TLY substrate, which provides low-loss characteristics ($\epsilon_{r}=2.2$, tanδ=0.0009) that are well suited for high-frequency operation. The substrate material was selected to minimize dielectric losses, improving the efficiency of electromagnetic wave propagation within the biological medium. As summarized in Table 2, the substrate and patch dimensions were carefully optimized to ensure impedance matching when in direct contact with the skin phantom. These refinements significantly reduce reflection losses, allowing for maximum energy transfer and increasing the reliability of glucose concentration detection. The patch antenna was fabricated using standard printed circuit board (PCB) etching techniques, as shown in Figure 4c. The compact size of the fabricated antenna, highlighted by the coin used for scale, emphasizes its portability and practicality for biomedical sensing applications. The combination of Double-L resonator topology, low-loss substrate selection, and precise dimensional optimization demonstrates improved sensitivity and enhanced detection capability, making the proposed antenna a strong candidate for noninvasive glucose monitoring applications.

**Figure 5:**
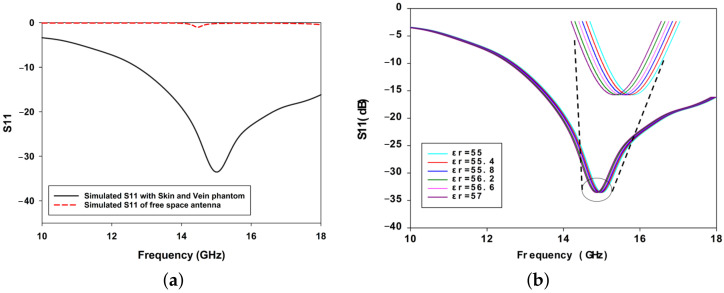
(**a**) Simulation and measurement of S11 parameters for the proposed microwave sensor and (**b**) simulated $S_{11}$ for various dielectric properties of the inserted vein.

As shown in Figure 5a, the sensor was designed and optimized to achieve matching with the skin phantom, enabling effective interaction with the embedded blood vessels. To investigate the impact of blood glucose level (BGL) variations on the sensor’s response, simulations were conducted by altering the relative dielectric constant of the blood vessels (veins) within the simulated skin phantom (Figure 5b). When the sensor was loaded with the phantom and injected with glucose solutions, the initial $S_{11}$ response was observed at 15.01 GHz. As the relative dielectric constant of the blood was increased from 55.2 to 56, corresponding to changes in glucose concentration, the sensor’s resonance frequency shifted to 14.830 GHz, resulting in a frequency shift of 180 MHz. This result highlights the sensor’s sensitivity to dielectric variations induced by BGL changes.

## 3. Experimental Results

### 3.1. Dielectric Properties of the Phantom Skin and Blood-Mimicking Glucose Solution

**Figure 6:**
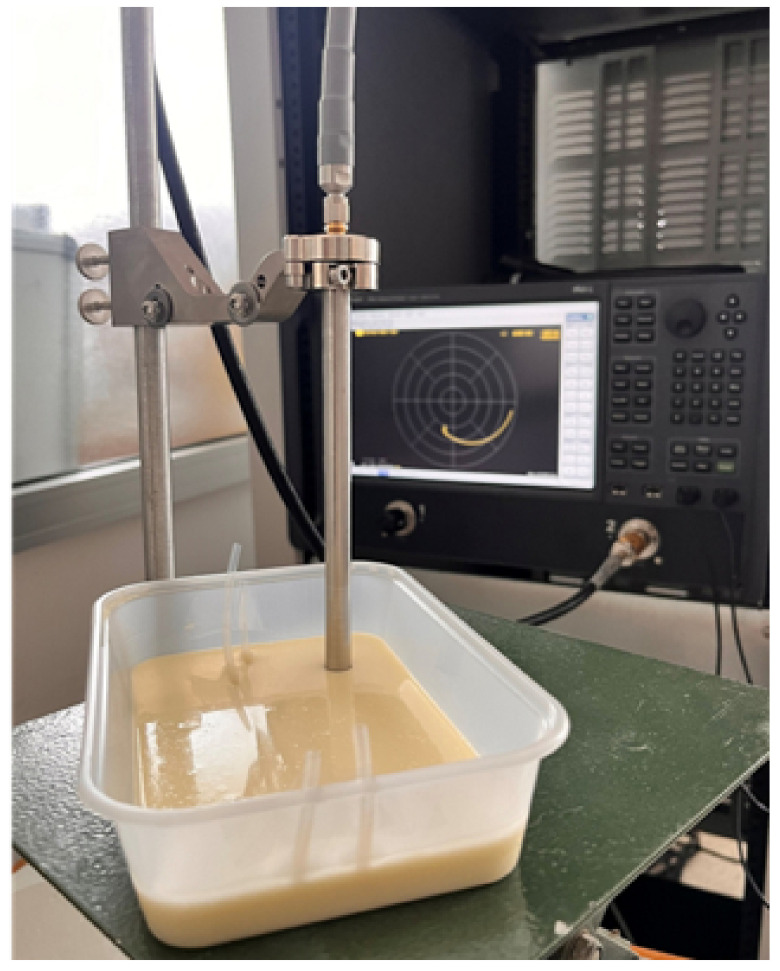
Dielectric properties of the skin phantom measured using a Keysight probe at 26 °C.

**Figure 7:**
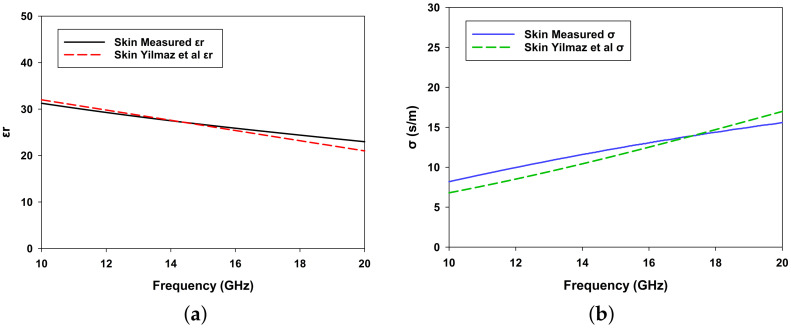
Comparison of measured and reported dielectric properties of the skin phantom. (**a**) measured relative permittivity ($\epsilon_{r}$) versus frequency compared with data from Yilmaz et al. [24] and (**b**) measured conductivity (σ) versus frequency compared with data from Yilmaz et al. [24].

To accurately measure the relative permittivity of the skin phantom within the 10 GHz to 20 GHz frequency range, we utilized an open-ended slim probe (Keysight N1501A-102) in conjunction with the PNA-L Network Analyzer N5234B from Keysight Technologies. Prior to measurement, the phantom mixture was allowed to cool and solidify completely in order to ensure stable material properties. The probe was then carefully positioned at the center of the phantom to achieve homogeneous and reliable results, following the procedure outlined in our previous [30,31,32]. The measurements were carried out in a controlled environment with a stable temperature of 26 °C and regulated humidity, effectively minimizing the influence of temperature fluctuations and external variations. Additionally, distilled water with a neutral pH was used in sample preparation to ensure consistency and prevent any alteration of the dielectric properties. Special attention was also given to probe contact, stray capacitance, and parasitic effects in order to maintain high measurement accuracy. This setup ensured accurate, repeatable, and reproducible dielectric property measurements across the frequency range of interest. Each measurement was repeated five times, after which the averaged data were used to minimize potential variability. The experimental setup employed for this process is illustrated in Figure 6. The measured dielectric properties, including both relative permittivity ($\epsilon_{r}$) and conductivity (σ), were then compared with values reported in [27] in order to evaluate the accuracy of the phantom. The comparison results are presented in Figure 7a,b.

The results show strong agreement with the reference study, with only minor discrepancies observed; specifically, the error in relative permittivity ranges from 1.2% to 2.5%, while the error in conductivity is slightly larger, ranging from 5% to 8%. These small deviations are likely due to unavoidable variations in the preparation process, slight differences in material properties, or environmental factors such as temperature stability during the experiments. Despite these minor mismatches, the measured properties confirm that the phantom reliably reproduces the dielectric behavior of human skin across the tested frequency range.

**Figure 8:**
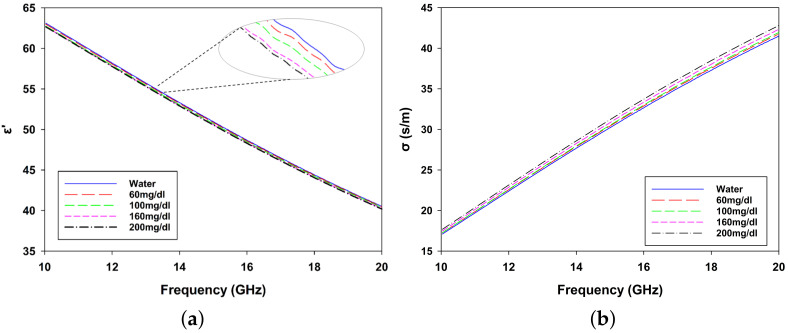
(**a**) Relative dielectric constant ($\epsilon_{r}$) and (**b**) conductivity (σ) of glucose solutions for concentrations of 60 mg/dL, 100 mg/dL, 160 mg/dL, 200 mg/dL, and water.

To further simulate physiological conditions, dielectric property measurements were extended to glucose solutions mimicking blood with varying glucose concentrations. The results for these solutions are presented in Figure 8a,b, providing further insights into the dielectric response of the system under different glucose levels. This comprehensive validation highlights the robustness of the phantom design and its suitability for evaluating microwave-based sensing systems.

### 3.2. Measurement Setup: Skin Phantom with Glucose Solutions Mimicking Physiological Conditions

**Figure 9:**
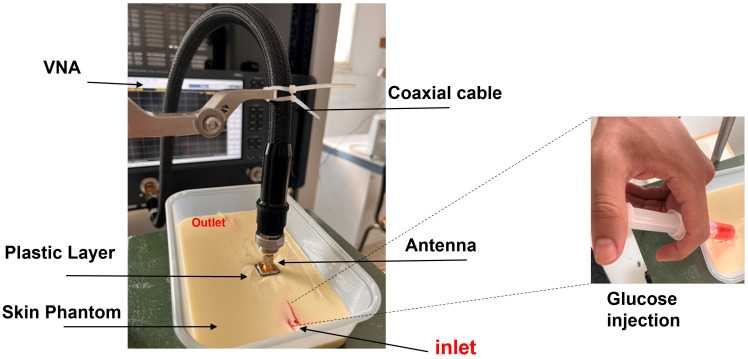
Experimental setup for microwave-based sensing on the brachial vein using a VNA with a syringe to inject glucose, mimicking blood flow in the vein.

Following the development and validation of the skin phantom and the dielectric property measurements of both the phantom and glucose solutions, the next step involved evaluating the performance of the antenna-based sensor under realistic conditions. The measurement setup was designed to replicate physiological interactions, with the sensor mounted directly on top of the skin phantom, as shown in Figure 9. By bridging the gap between simulation and experimentation, this setup allowed us to analyze the antenna’s sensitivity to changes in the dielectric properties induced by varying glucose concentrations. Solutions with different glucose concentrations ranging from 60 mg/dL to 200 mg/dL were prepared in separate syringes to mimic dynamic changes in physiological blood glucose levels. These solutions were injected sequentially into capillary-like channels embedded in the phantom, enabling controlled and reproducible testing conditions.

**Figure 10:**
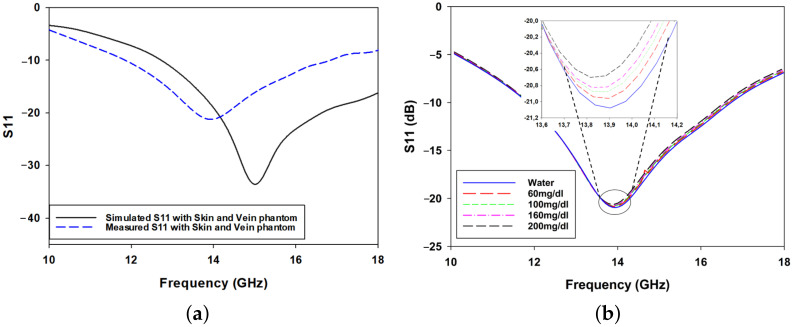
(**a**) Simulated and measured $S_{11}$ responses for the skin and vein phantom and (**b**) measured $S_{11}$ responses for different glucose concentrations: 60 mg/dL, 100 mg/dL, 160 mg/dL, 200 mg/dL, and water.

The $S_{11}$ response of the antenna was measured after matching the sensor to the skin phantom during simulations. The measurements were conducted using a PNA-L Network Analyzer (Keysight N5234B), which was calibrated using the Thru-Load-Open (TLO) method to minimize systematic errors and ensure accurate reflection coefficient measurements The measurements were performed under the same environmental conditions as those used for the skin phantom measurements, ensuring consistency in temperature and humidity control to minimize external variations. However, a noticeable shift in the measured $S_{11}$ response was observed compared to the simulated results, as shown in Figure 10a. This discrepancy can be attributed to several factors, including fabrication imperfections, material losses, and measurement uncertainties. Slight variations in substrate thickness, misalignment of layers, and imperfections in the etching or deposition process may have introduced deviations from the theoretical predictions. Additionally, surface roughness and dielectric losses which were not fully accounted for in the simulation model could have contributed to variations in the measured response. Another contributing factor is the presence of a thin plastic layer between the antenna and the skin phantom, which was absent in the simulations. While this layer affects impedance matching by slightly altering the dielectric properties, it serves an important role in protecting the antenna and maintaining the structural integrity of the phantom during repeated measurements.

To further investigate the effect of glucose concentration on the antenna’s response, a systematic measurement process was conducted. This experiment involved recording the $S_{11}$ response for each glucose concentration while ensuring careful replacement of the solution with one of higher concentration before proceeding to the next measurement. This process was repeated for all five concentrations. The measured responses reported in Figure 10b reveal a trend that closely follows the simulated response, demonstrating the sensor’s ability to detect variations in glucose concentration. Despite overall agreement between the measured and simulated trends, some frequency shift variations can be observed. One possible source of this discrepancy is the thickness of the skin layer poured over the channels, which was modeled to be precisely 1 mm in the simulations. Although meticulous care was taken during phantom preparation, slight deviations in this thickness may have affecting the measured response. Additionally, the presence of the plastic layer may have influenced the dielectric properties of the medium, further contributing to the observed differences between simulation and experiment.

To reduce these discrepancies and enhance accuracy in future studies, several improvements could be implemented. Refining the simulation models to account for surface roughness and material tolerances would lead to more precise predictions. Additionally, enhancing the fabrication process by incorporating more high-precision manufacturing techniques would help to minimize variations in material properties and structural dimensions. Finally, improving the measurement setup by using more precise calibration techniques and controlling environmental factors would contribute to more consistent and reliable experimental results.

Figure 10 presents the measured $S_{11}$ responses of the antenna-based sensor when the skin phantom with embedded veins is loaded with glucose solutions of varying concentrations (water, 60 mg/dL, 100 mg/dL, 160 mg/dL, and 200 mg/dL) reflecting normal human blood glucose ranges. As shown in the figure, an evident downward shift in the resonance frequency is observed as the glucose concentration increases. This behavior is primarily attributed to changes in the dielectric properties of the solution, particularly the rise in conductivity (σ) and subtle variations in relative permittivity ($\epsilon_{r}$). Higher glucose concentrations introduce more charge carriers, contributing to an increase in conductivity, which alters the wave propagation characteristics. In addition, the relative permittivity decreases slightly, influencing the resonance conditions of the antenna. The observed trend aligns with established electromagnetic theory, which suggests that an increase in medium conductivity leads to enhanced absorption and dispersion of electromagnetic waves, which in turn affects the sensor’s resonance properties. Furthermore, the broadening of the resonance dip at higher glucose concentrations suggests a possible increase in loss mechanisms, which could be linked to the dispersive behavior of glucose solutions at microwave frequencies [33]. These findings highlight the antenna’s sensitivity to glucose concentration variations, reinforcing its potential for practical noninvasive glucose monitoring applications.

**Figure 11:**
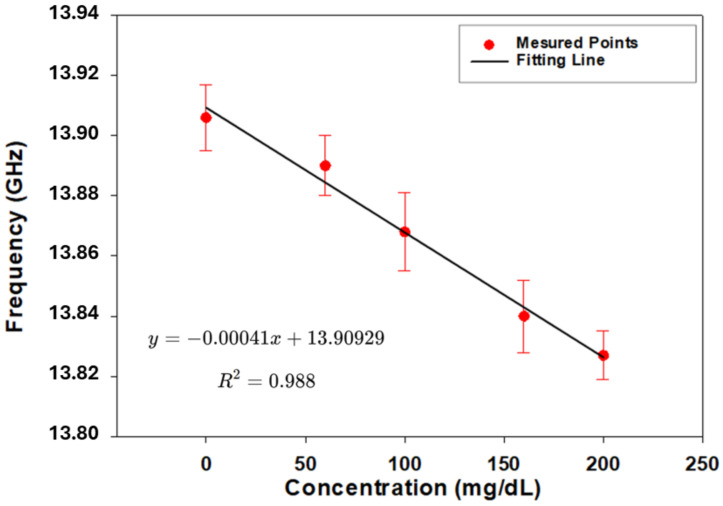
Frequency shift as a function of glucose concentration.

Figure 11 illustrates the linear relationship between glucose concentration and resonance frequency, demonstrating the sensor’s ability to detect glucose variations with high precision. The red points represent experimentally measured resonance frequencies, while the black line corresponds to the fitted linear regression, provided by the equation

(2) f=−0.00041C+13.90929,

where *f* is the resonance frequency in GHz and *C* is the glucose concentration in mg/dL. The high coefficient of determination $R^{2}=0.988$ confirms the strong correlation between glucose concentration and frequency shift. The presence of error bars on the measured data points accounts for the standard deviation observed in multiple repeated measurements, ensuring the consistency and repeatability of the sensor’s response. These variations highlight the robustness of the experimental setup and its minimization of uncertainties, further reinforcing the credibility of the results. The minimal deviation of the measured data from the fitted regression suggests that the sensor maintains high accuracy, making it a promising candidate for noninvasive glucose monitoring. Additionally, the sensor successfully differentiates between key glucose concentration ranges relevant to human physiology, including hypoglycemic levels (below 60 mg/dL), normal levels (72–108 mg/dL), and hyperglycemic levels (above 200 mg/dL). This ensures that the sensor mimics real physiological conditions, providing reliable and repeatable readings without the need for invasive blood sampling while reducing patient discomfort and infection risks. The linearity of the glucose–frequency relationship simplifies sensor calibration, making it well suited for real-time glucose monitoring applications. Future work could explore the impact of additional physiological factors such as temperature variations, pH levels, and interference from other biomolecules, which could help to further validate the sensor’s performance under real-world conditions. Incorporating these factors into the experimental framework would ensure the sensor’s long-term reliability and enhance its clinical applicability.

## 4. Conclusions

This study has demonstrated the feasibility of a microwave-based approach for noninvasive glucose monitoring using a skin-mimicking phantom with embedded vein-like channels. A miniature patch antenna was designed and optimized for operation at 14 GHz with a penetration depth of approximately 1.5 mm, with the results showing the antenna’s clear sensitivity to glucose concentration variations. Experimental measurements revealed a consistent downward shift in the resonance frequency as glucose levels increased, with a shift of approximately 80 MHz observed between the lowest (60 mg/dL) and highest (200 mg/dL) glucose concentrations. The strong linear correlation ($R^{2}=0.98$) between the resonance frequency and glucose concentration reinforces the reliability of the proposed sensing approach. While minor discrepancies between simulations and experiments were noted, likely due to fabrication tolerances and the addition of a protective plastic layer, the overall trends aligned well with theoretical expectations. This work highlights the potential of RF-based sensing for real-time noninvasive glucose monitoring. Future efforts will focus on refining sensor integration, enhancing calibration methods, and performing testing in more physiologically complex environments, with the goal of bringing this technology closer to clinical application and ultimately offering a more accessible and pain-free solution for diabetes management.

## Figures and Tables

**Table 1:** Amounts and percentages of materials used to create the skin-mimicking phantom.

Material	Amount (g)	Percentage (%)
Water	34	55
Gelatine	6	10
Oil	19	30
Soap	1.8	2.8
Salt	1.4	2.2

**Table 2:** Antenna dimensions.

Parameter	Description	Value (mm)
*W*	Width of the patch	8
*L*	Length of the patch	10
$W_{r}$	Width of the resonant arm	2.8
$L_{r}$	Length of the resonant arm	3
*C*	Feed gap spacing	0.1
$W_{H}$	Substrate width	15
$L_{H}$	Substrate length	15

## Data Availability

Data are contained within the article.
